# The Effectiveness of Online Exercise on Physical Activity, Motor Function, and Mental Health: Systematic Review and Meta-Analysis

**DOI:** 10.2196/64856

**Published:** 2025-08-15

**Authors:** Adelle Kemlall Bhundoo, Julian David Pillay, Jan Wilke

**Affiliations:** 1 Department of Basic Medical Sciences, Durban University of Technology, Durban, South Africa; 2 Institute of Sports Science, Department of Movement Sciences, University of Klagenfurt, Universitätsstrasse 65-67, Klagenfurt, 9020, Austria, 43 463 2700 6245

**Keywords:** online physical activity, digital exercise, physical wellbeing, mental wellbeing, motor performance, mental health.

## Abstract

**Background:**

Regular engagement in physical activity and exercise is associated with a multitude of physical and mental health benefits. Hence, it has been widely encouraged as a measure by which to combat somatic and psychological ailments. In view of the technical progress, the aging society and the public life restrictions issued during the COVID-19 pandemic, the delivery of interventions using digital devices has become highly popular.

**Objective:**

This systematic review and meta-analysis aimed to examine the effects of online exercise programs on physical activity (PA), motor performance, and mental health.

**Methods:**

Two independent investigators performed a systematic literature search, using PubMed, Cochrane Library, and Google Scholar. Randomized controlled trials assessing the effects of online exercise (OE) versus no exercise or face-to-face exercise (FFE) in healthy adults were included. Effect sizes (standardized mean difference [SMD]) were pooled using robust variance estimation. The quality of the included studies was assessed by 2 independent reviewers applying the PEDro scale, and publication bias was checked by means of funnel plots. To determine the certainty about the evidence, the results were rated by means of the GRADE (Grading of Recommendations, Assessment, Development, and Evaluations) criteria.

**Results:**

A total of 18 articles with moderate to high methodological quality (7/10 points on the PEDro scale), including a total of 3571 participants, were identified. Visual inspection of funnel plots provided indications of a publication bias for 2 out of 16 outcomes. According to the meta-analysis, OE was superior to no exercise regarding strength (SMD=0.61, 95% CI 0.06 to 1.15, n=5 studies), balance (SMD=0.52, 95% CI 0.06 to 0.99, n=4 studies), endurance (SMD=0.85, 95% CI −0.01 to 1.70, n=5 studies), PA (SMD=0.46, 95% CI 0.05 to 0.87, n=5 studies), depression (SMD=1.08, 95% CI −0.01 to 2.16, n=4 studies), mood or emotion (SMD=0.47, 95% CI 0.05 to 0.90, n=5 studies), mental well-being (SMD=0.79, 95% CI 0.06 to 1.52, n=4 studies), and self-efficacy (SMD=1.1, 95% CI 1.03 to 1.17, n=3 studies). Compared to FFE, OE was noninferior (*P*>.05) except for gait speed which improved more following FFE (SMD=0.25, 95% CI 0.24 to 0.26, n=2 studies). The certainty about the evidence (GRADE criteria) was low to moderate for all comparisons.

**Conclusions:**

OE represents an effective strategy to improve PA, physical function, and mental health in healthy adults and may hence help combat physical inactivity. However, despite the encouraging findings, some limitations need to be tackled before drawing definitive conclusions. These, inter alia, include a small total number of studies and substantial between-trial heterogeneity for some outcomes. Furthermore, as this review focused on healthy adults, future research examining other populations (eg, children and adolescents) is needed.

## Introduction

Physical activity (PA) has been demonstrated to represent a significant contributor to health [1]. Irrespective of the type of PA, its advantages are unanimously agreed upon as regular movement has been shown to decrease morbidity and improve prognoses in relation to a wide variety of pathologies [2,3]. Regular PA is, furthermore, linked to improved psychological well-being [4,5] and a lower incidence of mental disorders [6]. In recent years, however, the increasing time demands placed on the average individual have posed a definite threat to the ability to achieve the recommended levels of PA [7]. This is of utmost importance because sedentary lifestyle habits are a risk factor for major noncommunicable diseases, such as diabetes, obesity, and hypertension [8-10] as well as for mental health conditions such as depression and anxiety [11].

In view of the pivotal role of PA, the availability of exercise offers (eg, gyms, sports clubs, and public exercise facilities) is central to maintaining public health. Yet, as the hallmark of the demographic change, the number of older people is expected to more than double within the next 25 years [12], meaning that more and more seniors with comorbidities will require attention, supervision, and treatment. Of note, PA decreases with increasing age, which reflects a pressing need for novel and easily accessible PA platforms [13]. Further to this, there has been a shift toward individualization in both society and health care [14,15]. All these issues highlight that exercise interventions should be tailored according to the preferences of the individual [15,16].

The COVID-19 pandemic has been another accelerator to the development of digital exercise offers. The global population was denied access to exercise establishments in an attempt to curb the spread of the virus [17], which inadvertently forced otherwise physically active people into a state of inactivity [18,19]. This created a large demand for online programs that were user-friendly, effective, and, most importantly, allowed individuals from various fitness backgrounds to remain physically active without increasing the risk for contracting COVID-19 [20]. A study investigating the adaptability of digital exercise during crises indicated that these platforms have the ability to increase PA and control weight gain during instances where regular PA cannot be accessed [21]. Another study indicated that the use of online PA tools can assist in improving PA, thus eliciting physical and mental health benefits during the COVID-19 pandemic and any future pandemics of similar nature [22]. This adaptability provides the basis for the inclusion of online PA engagement in the protocols and planning for future pandemics and pandemic-like crises [21].

Online exercise programs have included simple pedometer tracking software, PA support forums, home exercise guidelines, PA reporting logs, structured virtual exercise platforms, and live-streamed workouts [23]. Alike programs have remained popular even though pandemic-related restrictions have been lifted. As vaccinations have formed a foundation for infection control, the virus continues to mutate, leaving room for future outbreaks that cannot be controlled without adjustments to the current vaccines, which may still require the need for social distancing in the future [24,25]. Hence, the necessity for efficient online exercise programs remains relevant.

The research on online PA during the COVID-19 pandemic yielded many beneficial results; however, there are some noteworthy challenges and barriers that need to be addressed in forthcoming studies. One study concluded that some older participants lacked technological skills that may be necessary for optimal online PA participation, whereas others lacked the financial capacity to fully engage with online PA resources [25]. The unavailability of sufficient internet access and digital technology infrastructures was highlighted as an additional challenge, especially in rural and outlying communities [26]. A different study indicated that a lack of participant confidence and a concern for risks may present as barriers to effective online PA interactions [27]. Studies further suggest that populations such as individuals that are visually impaired can struggle to access these programs, thus reducing access to PA benefits [28].

Two previous systematic reviews [23,26] found beneficial effects of online PA programs on a variety of outcomes. However, more than 10 years have passed since their publication. Furthermore, these articles either did not include a meta-analysis [23], combined healthy and diseased individuals [23,26], or did not report on possible changes in mental health [23,26]. Previous systematic reviews have demonstrated that digital health interventions and structured exercise programs are effective in managing chronic diseases such as type 2 diabetes (T2D). For example, one review [29] systematically reviewed digital health technologies for T2D management, highlighting their potential in improving patient outcomes [29]. Another review emphasized the effectiveness of exercise programs in enhancing health outcomes for individuals with T2D diabetes, reinforcing the critical role of structured exercise in chronic disease management [30].

However, despite these advancements, there remains a significant literature gap regarding the application of these findings to healthy individuals, particularly in the context of online exercise programs aimed at improving physical activity and mental health. This systematic review aims to address this gap by evaluating the effectiveness of online exercise interventions specifically for healthy individuals, thus providing a comprehensive understanding of their potential benefits in this population.

## Methods

### Overview

We performed a systematic review and meta-analysis summarizing the effects of digital online exercise (OE) versus no exercise (NEX) or conventional face-to-face exercise (FFE). Outcomes included markers of PA, physical function, and mental health. The review protocol was prospectively registered in the International Prospective Register of Systematic Reviews (PROSPERO) database (CRD42022338871). The PRISMA abstract checklist (Checklist 1) and PRISMA checklist (Checklist 2) were used to ensure that reporting was compliant.

### Date Sources and Source Strategy

Two independent investigators (AKB and JW) performed separate individual systematic literature searches using PubMed, Cochrane library, and Google Scholar. Searches were performed initially in August 2022 and updated in February 2024. A uniform search term was used to conduct the literature search across all 3 search engines, the term was: “(exercise OR “PA”) AND (internet* OR online* OR web* OR e-health OR digital OR tele* OR virtual) AND home*.” Searches were limited to articles published from January 1, 2000 (Multimedia Appendix 1). The decision to start the range at the year 2000 was made to accommodate for the lack of the necessary online technologies. Such technologies include but are not limited to high-speed internet, streaming platforms, and advanced audio-visual hardware required for effective and interactive online exercise programs. In addition to database searches, the reference lists of all included studies were checked to identify further potentially eligible articles [31]. Articles that met the initial screening criteria were entered into a Microsoft Excel spreadsheet for title and author screening. Duplicate and suspected duplicate articles were individually downloaded and compared. At this stage, if an article was confirmed as a duplicate, it was removed from the spreadsheet.

### Eligibility Criteria

Studies were considered eligible for inclusion based on the following criteria (Textbox 1).

Textbox 1. **Inclusion criteria**

- Randomized controlled trial design.
- Online exercise intervention with a duration of at least 4 weeks.
- Recruitment of healthy adults (18 y of age and older with no diagnosed chronic illness that may impact engagement with the exercise programs or influence the benefits or risks associated with increases in exercise engagement).
- Measurement of physical activity, motor performance, and mental health surrogates.
- Publication in English language.

Eligibility checks were performed by two independent investigators (AKB and JW), and disagreements were resolved by discussion.

### Study Quality, Risk of Bias, and Certainty About the Evidence

Study quality was evaluated by means of the PEDro scale [32,33]. Two examiners (AKB and JDP) independently rated each study, and in case of disagreement, meetings were held to achieve consensus [34]. For the identification of a potential reporting bias, we used visual inspection of funnel plots. To classify the certainty about the evidence as very low, low, moderate, or high, the criteria of the GRADE (Grading of Recommendations, Assessment, Development, and Evaluations) working group were applied (Multimedia Appendix 2 [35]). Briefly, the evaluation starts with the assumption of a high certainty about the evidence due to the randomized controlled trial design. The GRADE framework then suggests adjusting the certainty as follows: in case of risk of bias, imprecision, inconsistency of results, indirectness of the evidence, or publication bias, one point is subtracted for each weakness. In contrast, a large magnitude of the effect or a dose-response gradient leads to an upgrade of the certainty rating [35].

### Data Extraction

We extracted the following data: sample size, participant characteristics, interventions, measurements, and results (pre-post changes plus SDs of each intervention arm). The primary outcomes of the meta-analysis were PA, motor function (strength, endurance, balance, flexibility, gait, and body composition), and mental health (depression, anxiety, mood or emotion, psychological well-being, sleep, and self-efficacy). If a study performed more than one test assessing the same outcome, all effect sizes (ES) were obtained.

### Data Processing and Statistical Analysis

For each trial arm, we extracted the mean baseline and postintervention values, pre-post changes, and the related SD. In case of incomplete reporting (ie, missing SDs of the changes from baseline), the corresponding authors of the trials were contacted. If no values could be obtained, missing data were determined from figures or imputed according to Cochrane recommendations:


(1) $$SD_{change}=\sqrt{SD_{baseline}^{2}+SD_{postintervention}^{2}-(2\times Corr\times SD_{baseline}^{2}\times SD_{postintervention}^{2})}$$

where Corr=0.7. The value chosen for Corr represents a conservative estimate of the correlation between the baseline and posttreatment SDs [36].

Robust variance estimation random [37] was used to pool the standardized mean differences (SMD) and 95% CI for OE versus NEX as well as OE versus FFE. Dependency of ES was considered by nesting the term “study” as a random factor in the model. The between-study variance component was determined by means of Tau2, using the method-of-moments estimate; for within-study variance (more than one dependent effect size), ω2 was calculated [38]. We interpreted the resulting ES as follows: small (SMD=0.2), medium (SMD=0.5), or large (SMD=0.8). The used software was R (R Foundation for Statistical Computing, Vienna, Austria), packages meta (G Schwarzer) and robumeta (version 2.0) [39].

### Ethical Considerations

Ethical approval for this study was applied for and obtained before the commencement of data collection. Approval was issued by the Institutional Research Ethics Committee (IREC) at the Durban University of Technology (DUT; IREC 090/20).

## Results

### Overview

**Figure 1.**
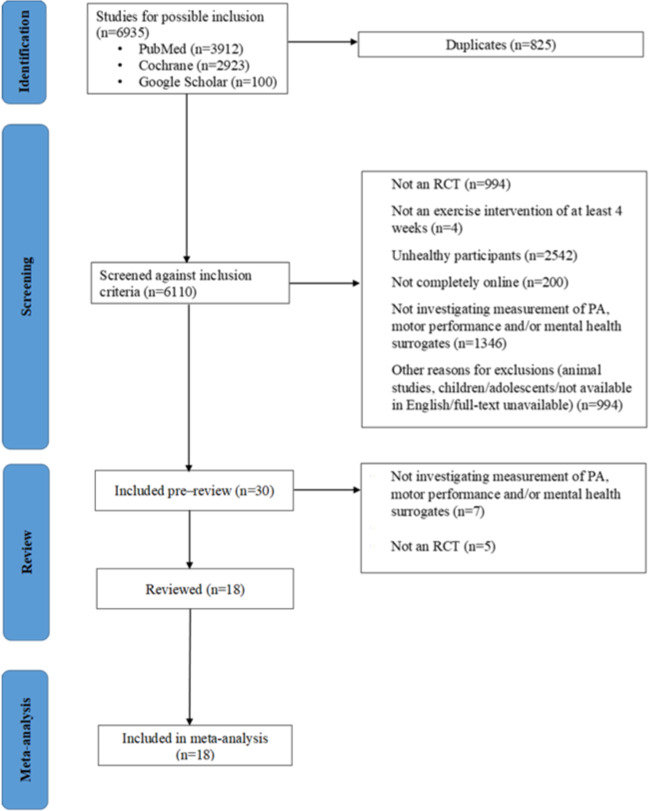
Flow of the literature search. PA: physical activity; RCT: randomized controlled trial.

The initial database searches yielded a total of 6335 articles (Figure 1). After removal of duplicates and application of inclusion criteria, 18 studies were included.

### Study Characteristics

The characteristics of the 18 included studies, which included a total of 3531 cumulative participants, are displayed in Table 1. Half (n=9) of the studies had participants that were older than 65 years, the remaining half (n=9) examined young or middle-aged adults. Five studies compared OE to FFE, while 12 investigated OE versus NEX, and one compared OE to both FFE and NEX. Motor performance outcomes were assessed in 11 studies, PA was measured in 5 studies, and mental health was examined in 8 studies. The mean (SD) duration of the OE interventions was 12.5 (10.34) weeks and the mean (SD) training frequency was 2.7 (1.94) sessions per week.

**Table 1.** Study Characteristics.

Study		Sample	Intervention	Outcomes
Online exercise versus inactive control				
	Napolitano et al (2003) [40], n=65	9M^a^, 56W^b^; mean 43 (SD 10) years	Group 1: online PA^c^ material including weekly health and PA-related tip sheets; 12 wk Group 2: no exercise for 12 wk	PA
	Marcus et al (2016) [41], n=205	INT^d^: 104 W; mean 39 (SD 11) years CON^e^: 101 W; mean 40 (SD 10) years	Group 1: online PA material, combined with goal setting, and motivational messaging; 6 mo Group 2: received general health tips such as diet, etc; no PA material; 6 mo	PA, depression, mood or emotion, and self-efficacy
	Dekker-van Weering et al (2017) [42], n=36	INT: 12 W, 9 M; mean 71 (SD 4) years CON: 10 W, 5 M; mean 69 (SD 4) years	Group 1: live-streaming exercise, 3× 30 min/week; 12 wk Group 2: no exercise for 12 wk	Mental wellness
	Hartman et al (2017) [43], n=205	INT: 104 W; mean 39 (SD 11) years CON: 101 W; mean 40 (SD 10) years	Group 1: accelerometer tracks PA combined with goal setting and motivational messaging; 6 mo Group 2: received general health tips such as diet, etc; no PA advice; 6 mo	PA
	Beauchamp et al (2021) [44], n=241	187 W, 54 M; mean 73 (SD 5) years	Group 1: live-streaming, group sessions with a trainer, and social breakout groups, 50‐60min/session for at least 3× weekly; 12 wk Group 2: live-streaming, individual sessions with a trainer, 50‐60 min/session at least 3× weekly; 12 wk Group 3: no exercise for 12 wk, access to program at the end of 12 wk	Depression and mental wellness
	Yi and Yim (2021) [45], n=70	INT: 8 M, 27 W; mean 76 (SD 6) years CON: 4 M, 31 W; mean 77 (SD 6) years	Group 1: live-streaming exercise, 2× 40 min/week, 8 wk Group 2: no exercise for 8 wk	Strength, endurance, balance, and gait speed
	Chang et al (2022) [46], n=73	56 W, 17 M; mean 70 (SD 5) years	Group 1: live-streaming exercise, 70‐90 min/session, 8 wk Group 2: no exercise for 8 wk	Strength, endurance, balance, and flexibility
	Langeard et al (2022) [47], n=41	OE^f^: 10 W, 3 M; mean 73 (SD 4) years FFE^g^: 9 W, 6 M; mean 72 (SD 4) years CON: 8 W, 5 M; mean 74 (SD 4) years	Group 1: live-streaming with trainer, 2× 60 min/week; 16 wk Group 2: face-to-face with same trainer, 2× 60 min/week; 16 wk Group 3: no exercise for 16 wk	Strength, endurance, and body fat
	Tekin and Cetisli-Korkmaz (2022) [48], n=255	INT: 72 M, 60 W; mean 68 (SD 4) years CON: 66 M, 57 W; mean 70 (SD 5) years	Group 1: online recorded videos, 5days/week, participants recorded themselves doing exercise and forwarded to researchers; 4 wk Group 2: no exercise for 4 wk	Strength, balance, gait speed, depression, and self-efficacy
	Waden and Cartwright (2022) [49], n=34	INT: 17 W; mean 43 (SD 11) years CON: 14 W, 3 M; mean 42 (SD 10) years	Group 1: live-streaming yoga program, 2×‐3× 50 min/week; 6 wk Group 2: no exercise for 6 wk, access to exercises post intervention	Depression, anxiety, mood or emotion, mental wellness, and self-efficacy
	Wilke et al (2022) [50], n=763	237 M, 523 W; 2 D^h^, 1 U^i^; mean 33 (SD 13) years	Group 1: live-streaming synchronous exercise program, with trainer, according to schedule, 30‐60 min/session, 5 days/week, 4 wk, recorded sessions 4 wk post intervention Group 2: no exercise for 4 wk, access to recorded sessions 4 wk post intervention	PA, anxiety, mood/emotion, mental wellness, and sleep
	Wu et al (2022) [51], n=80	34 W, 46 M; mean 23 (SD 3) years	Group 1: live-streaming exercise, 3× 30 min/week, 4 wk Group 2: no exercise for 4 wk	Endurance, mood or emotion, and sleep
	Zengin Alpozgen et al (2022) [52], n=30	INT: 10 W, 5 M; mean 67 (SD 4) years CON: 7 W, 8 M; mean 69 (SD 6) years	Group 1: live-streaming sessions with trainer, 3×40‐45 min/week; 6 weeks Group 2: no exercise for 6 wk, access to program at the end of 6 wk	Strength, endurance, balance, flexibility, and PA
Online exercise versus active control				
	Marcus et al (2007) [53], n=249	OES^j^: 68 W, 14 M; mean 46 (SD 9) years OET^k^: 66 W, 15 M; mean 45 (SD 9) years FFE: 72 W, 14 M; mean 45 (SD 10) years	Group 1: online PA material with PA logs only Group 2: online PA material with PA logs, goal setting, motivational messaging, and personalized feedback Group 3: face-to-face PA material with PA logs, goal setting, motivational messaging, and personalized feedback	Endurance
	Pressler et al (2010) [54], n=105	12 W, 93 M; median age 48 (range 25‐60) years	Group 1: online structured exercise, 3×30‐70min/week; 12 weeks Group 2: online unstructured exercise, no schedule, no specified sessions/week; 12 wk	Endurance, blood pressure, and body fat
	Baez et al (2017) [55], n=37	27 W, 9 M; mean 71 (SD 6) years	Group 1: online personalized program with social environment for group exercising, messaging and persuasion features, 2×30‐40min/week; 8 weeks Group 2: home-based program, no social or individual persuasion features, 2×30‐40min/week; 8 weeks	Strength, gait speed, mood, or emotion
	Jennings et al (2020) [56], n=1048	INT: 247 M, 31 W; mean 75 (SD 7) years CON: 700 M, 70 W; mean 74 (SD 7) years	Group 1: live-streaming therapist-led exercise, 1‐9 sessions/week; 5 wk Group 2: no therapist-led exercise for 5 wk	Strength and gait speed
	Kikuchi et al (2021) [57], n=34	INT: 12 M, 11 W; mean 45 (SD 16) years CON: 3 M, 8 W; mean 39 (SD 10) years	Group 1: live-streaming, 2×60 min/week; 8 wk Group 2: face-to-face, 2×60 min/week; 8 wk	Strength and blood pressure
	Langeard et al (2022) [47], n=41	OE: 10 W, 3 M; mean 73 (SD 4) years FFE: 9 W, 6 M; mean 72 (SD 4) years CON: 8 W, 5 M; mean 74 (SD 4) years	Group 1: live-streaming with trainer, 2×60 min/week; 16 wk Group 2: face-to-face with same trainer, 2×60 min/week; 16 wk Group 3: no exercise for 16 wk	Strength, endurance, and body fat

a M: men.

b W: women.

c PA: physical activity.

d INT: intervention.

e CON: control.

f OE: online exercise.

g FFE: face-to-face exercise.

h D: diverse.

i U: unspecified.

j OES: online exercise standard.

k OET: online exercise tailored.

### Study Quality and Risk of Reporting Bias

Ratings on the PEDro scale ranged from 3 to 10 with a mean of 7.2 (SD 2.3) out of 10 points (Table 2).

All studies reported between-group statistical analyses for at least one key outcome measure. Almost all (94%, n=17) articles provided clear eligibility criteria; had intervention and control groups that were similar at baseline with regards to key prognostic indicators; and reported point and variability measures for at least one key outcome measure. A clear majority (83%, n=15) used randomized groups allocation, while 78% (n=14) ensured concealed group allocations. A slightly smaller share (72%, n=13) reported all participants receiving the intended intervention and the use of intention to treat in cases where participants did not receive the intended interventions; and reported outcome measures for at least 85% of the initial group allocations. In terms of blinding, 61% (n=11) of the studies reported participant blinding, 44% (n=8) indicated assessor blinding, and only 17% (n=3) stipulated therapist blinding.

Visual inspection of funnel plots (Multimedia Appendix 3) yielded indications for a reporting bias regarding strength measures in OE versus FFE and for mood or emotion in OE versus NEX.

**Table 2.** PEDro scores of the included studies.

	Baez et al (2017) [55]	Beauchamp et al (2021) [44]	Chang et al (2022) [46]	Dekker-van Weering et al (2017) [42]	Hartman et al (2017) [43]	Jennings et al (2020) [56]	Kikuchi et al (2021) [57]	Langeard et al (2022) [47]	Marcus et al (2007) [53]	Marcus et al (2016) [41]	Napolitano et al (2003) [40]	Pressler et al (2010) [54]	Waden and Cartwright (2022) [49]	Wilke et al (2022) [50]	Wu et al (2022) [51]	Yi and Yim (2021) [45]	Zengin Alpozgen et al (2022) [52]
Eligibility criteria specified	1^a^	1	1	1	1	1	0^b^	1	1	1	1	1	1	1	1	1	1
Random group allocation	1	1	0	1	1	0	0	1	1	1	1	1	1	1	1	1	1
Allocation concealed	1	1	0	1	1	0	0	1	1	1	1	1	0	1	1	1	1
Groups similar at baseline regarding most important prognostic indicators	1	1	1	1	1	1	1	1	1	1	0	1	1	1	1	1	1
Participant blinding	1	1	1	0	1	0	0	1	1	1	1	1	0	1	0	0	0
Therapist blinding	0	1	0	0	0	0	0	1	0	1	0	0	0	0	0	0	0
Assessor blinding	1	1	0	0	1	0	0	1	0	1	0	0	0	1	0	0	1
Measures of at least one outcome obtained from>85% of participants	1	1	0	1	1	0	0	1	1	0	1	1	1	1	1	1	1
All participants received treatment or control as allocated or data for >1 outcome analyzed intention to treat	1	1	1	1	1	0	0	1	1	1	1	0	0	1	1	0	1
Results of between-group comparisons reported for >1 outcome	1	1	1	1	1	1	1	1	1	1	1	1	1	1	1	1	1
Point measures and measures of variability for >1 outcome	1	1	1	1	1	0	1	1	1	1	1	1	1	1	1	1	1
TOTAL PEDro Score	9	10	5	7	9	2	3	10	8	9	7	7	5	9	7	6	8

a 1=Yes.

b 0=No.

### Meta-Analysis

Compared to NEX (Table 3), OE had a moderate to large beneficial effect on measures of strength (SMD=0.61, 95% CI 0.06-1.15, *P*=.04; GRADE: moderate certainty about the evidence), balance (SMD=0.52, 95% CI 0.06-0.99, *P*=.04; moderate certainty), endurance (SMD=0.85, 95% CI −0.01 to 1.70, *P*=.05; low certainty), and physical activity (SMD=0.46, 95% CI 0.05-0.87, *P*=.04; moderate certainty). No difference was found for gait speed (moderate certainty) and flexibility (low certainty).

**Table 3.** Effects of online exercise versus inactive control on markers of motor performance and physical activity.

Outcome	Studies, n (ES^a^)	SMD^b^ (95% CI)	*P* value	τ^2^ / ω^2^	GRADE^c^ rating
Strength	5 (12)	0.61 (0.06 to 1.15)	.04	0.14/0	Moderate
Endurance	5 (6)	0.85 (–0.01 to 1.70)	.05	0.32/0	Moderate
Balance	4 (9)	0.52 (0.06 to 0.99)	.04	0.13/0	Moderate
Gait	2 (3)	0.26 (–2.23 to 2.75)	.41	0.03/0	Moderate
Flexibility	2 (4)	0.74 (–6.32 to 7.80)	.41	0.53/0	Moderate
Physical activity	5 (7)	0.46 (0.05 to 0.87)	.04	0.07/0	Moderate

a ES: effect size.

b SMD: standard mean difference.

c GRADE: Grading of Recommendations, Assessment, Development, and Evaluations.

With regard to mental health (Table 4), OE was moderately to strongly superior to NEX for depression (SMD=1.08, 95% CI −0.01 to 2.16, *P*=.05; moderate certainty), mood/emotion (SMD=0.47, 95% CI 0.05-0.90, *P*=.04; low certainty), mental well-being (SMD=0.79, 95% CI 0.06-1.52, *P*=.05; moderate certainty), and self-efficacy (SMD=1.1, 95% CI 1.03-1.17, *P*=.06; moderate certainty). No effect was found for anxiety and sleep (*P*>.05, moderate certainty).

**Table 4.** Effects of online exercise versus inactive control on markers of mental health.

Outcome	Studies, n (ES^a^)	SMD^b^ (95% CI)	*P* value	τ^2^ / ω^2^	GRADE^c^ rating
Depression	4 (5)	1.08 (–0.01 to 2.16)	.05	0.07/0.4	Moderate
Anxiety	2 (2)	0.20 (–3.19 to 3.59)	.59	0.10/0	Moderate
Mood or emotion	5 (10)	0.47 (0.05 to 0.90)	.04	0.08/0	Moderate
Mental well-being	4 (5)	0.79 (0.06 to 1.52)	.04	0/1.4	Moderate
Sleep	2 (3)	–0.26 (–2.57 to 2.06)	.40	0.05/0	Moderate
Self-efficacy	3 (3)	1.1 (1.03 to 1.17)	.06	0/0	Moderate

a ES: effect size.

b SMD: standard mean difference.

c GRADE: Grading of Recommendations, Assessment, Development, and Evaluations.

Regarding OE versus FFE (Table 5), no difference was found for most measures (strength: very low certainty, endurance: moderate certainty, body fat: low certainty). However, gait speed improved slightly more in OE (SMD=0.25, 95% CI 0.24-0.26, *P*=.002; moderate certainty).

**Table 5.** Effects of online exercise versus face-to-face exercise on markers of motor performance and body composition.

Outcome	Studies, n (ES^a^)	SMD^b^ (95% CI)	*P* value	τ^2^ / ω^2^	GRADE^c^ rating
Strength	4 (14)	–0.20 (–0.84 to 0.45)	.41	0.13/0	Low
Endurance	3 (4)	–0.04 (–0.34 to 0.27)	.66	0/0	Moderate
Gait speed	2 (2)	0.25 (0.24 to 0.26)	.002	0/0	Moderate
Body fat	2 (2)	–0.07 (–4.87 to 4.73)	.88	0.18/0	Low

a ES: effect size.

b SMD: standard mean difference.

c GRADE: Grading of Recommendations, Assessment, Development, and Evaluations.

## Discussion

### Principal Findings and Comparison to Previous Work

The manifold benefits of exercise have been repeatedly presented in the literature [1,2]. Recently, there has been a shift toward the use of online platforms aiming to provide easily accessible exercise opportunities to the general population [23,26]. To the best of our knowledge, this article is the most comprehensive and up-to-date quantitative summary of the available evidence. We show that OE is non-inferior to FFE and superior to NEX in a variety of measures of physical function, mental health, and PA. Regarding motor performance, OE induced moderate-to-large improvements in strength, endurance, and balance, which accords with data from older reviews [23,26]. In contrast, gait speed and flexibility did not change. This may be related to a lack of power as both outcomes were investigated in only two studies. For mental health outcomes, the very large positive effects on depression (SMD=1.08) and self-efficacy (SMD=1.1) were striking. A beneficial impact, although of smaller magnitude, was also observed for mood or emotion and mental well-being. While the sizable effects of OE on most psychological outcomes are in line with studies examining face-to-face exercise [58,59], the lack of a change in anxiety is a bit surprising because anxiety had been reported to improve in response to conventional exercise [60]. Again, the nonsignificant effect size may be due to the small number of studies (n=2).

### Clinical Implications

Our results have a variety of practical implications. It would be reasonable to assume that exercise performed face-to-face would be the most effective way to improve health and performance. Yet, as we demonstrated noninferiority of OE regarding most outcomes, the selection of the exercise mode may be left to the individual preference. This is particularly relevant because under some circumstances, OE may be the more feasible option. For example, there is a distinct need for the development and implementation of innovative and convenient exercise strategies to combat physical inactivity, and some individuals living in scarcely populated areas may have difficulties finding FFE. Also, engagement in OE may save travel time and costs. The use of OE can hence deliver an opportunity within certain populations where inactivity is rife [61]. A study showed that digital PA programs can be integrated into workplace interfaces to address inactivity that occurs during work hours [62].

Half of the studies reviewed (n=9) used older participants (>65 years). Of note, the projected change in demographics leaves older individuals at a higher risk for decreased PA [13]. Research suggests that the age and PA are inversely proportionate in that an increase in age tends to result in decreased levels of PA. This has been attributed to several factors, decreased physical capability, socioeconomic barriers, and a lack of social support [63]. For this particular population, the use of online PA can potentially increase engagement as individuals could access programs remotely [64]. Thus, OE increases the potential to provide these older adult persons with a safe, effective, and accessible PA opportunity. As much as there is the limitation of digital literacy [65], research suggests that this limitation is improving [65]; hence, as the years unfold, the use of online PA interventions may become more beneficial and used in the older population [66].

As indicated, OE may be of particular interest in rural areas where public PA infrastructure (eg, availability of sports clubs, or gyms) is limited [67]. In these instances, the distance between residential dwellings and traditional PA establishments is too large for individuals to travel [68]. Hence, online PA programs, especially in light of growing internet accessibility throughout the world, could offer the benefits of PA without the need for increased traveling [69]. As the share of users with internet access is increasing steadily around the globe [70], OE may be of help in the fight against the pandemic of inactivity.

Our results on mental health outcomes showed that OE was superior to NEX for depression, mood/emotion, mental well-being, and self-efficacy. This implies that OE could be of value in cases of mental illness where PA facilities are either unavailable or undesired by patients. Further to this, in a clinical setting, OE incorporating group interactions into the structure of the platform can present the opportunity for social interaction [71]. This adds a social aspect to the benefits of online PA [72]. The use of social integrations in online PA programs allows for individuals with similar backgrounds, such as those with mental illness, to interact [73]. Thus, increasing motivation and desire to remain consistent with OE, which enhances long-term program engagement [74].

Finally, OE can also help to reduce the direct costs of exercise as streamed contents would theoretically be available to an unlimited number of individuals rather than being limited to those who can afford memberships in face-to-face PA facilities [75]. However, some caveats also require consideration. A large benefit of FFE is that coaches have a 3D view on exercising individuals, which allows for an easier correction and monitoring of movement execution. While this is less a concern for healthy individuals, it may become relevant for older adults or people with disease [76,77]. Another issue relates to interpersonal factors, as sports and exercise cannot be reduced to their direct physical effects. When exercising in a group, social interaction can increase motivation or build friendships that could be of value in other aspects of life [78,79].

### Strengths and Limitations

A particular strength of our review is that we only included randomized controlled trials, as this study type is viewed to be the most valid in the assessment of intervention effectiveness [80]. In addition, the mean PEDro score of 7.2 indicates a good overall quality, which adds to the validity of the findings [50]. A further strength was the use of GRADE ratings to assess the quality of evidence analyzed in this manuscript. This adds to the overall quality of the review by creating a more accurate representation of data. Finally, to reduce heterogeneity, we limited inclusions to studies with healthy participants. There are certain limitations identified within our study that should be taken into consideration in the planning and execution of any future studies. Firstly, our study used an identical search across three different search engines. Although this is in keeping with regular review protocols, future studies may benefit from the use of additional search engines to ensure that results are extensive and exhaustive. Secondly, the filter applied to exclude studies published before January 2000 was included to streamline the search results; however, it may have resulted in exclusion of some relevant data. Hence, we recommend that future reviews use wider timeframes. Thirdly, in about one third of the outcomes, the certainty about the evidence was low, which was probably due to the small number of studies resulting in large confidence intervals, reporting bias, and heterogeneity. We therefore reinforce the need for additional studies to fully gauge the long-term effects of OE [41,53,54]. This particularly applies because some of the studies included in our review were conducted during the COVID-19 pandemic, and it is unknown how the specific conditions affected the overall result of the meta-analysis. It is imperative that more studies are conducted in a less volatile period to observe unbiased effects in relation to the everyday lives of individuals. Fourth, the search criteria did not allow for online PA interventions that assessed mental outcome measures only and instead focused on a coupling of physical and mental outcome measures. Further investigations are required to report specifically on the effectiveness of online PA intervention on mental health, against active controls and inactive controls. Finally, our study included only healthy participants, although this was specifically conducted to encourage heterogeneity. Further investigations are required to report specifically on the effectiveness and safety of OE in unhealthy participants.

### Conclusions

There is mostly moderate certainty evidence that OE improves PA, motor performance, and mental health when compared to NEX. In addition, OE seems non-inferior to FFE. However, additional studies including larger sample sizes, longer study durations, and long-term follow-up measurements are warranted in order to better delineate the benefits of OE interventions.

## Supplementary material

10.2196/64856 Multimedia Appendix 1 Article search strategy.

10.2196/64856 Multimedia Appendix 2 GRADE (Grading of Recommendations, Assessment, Development, and Evaluations) classification.

10.2196/64856 Multimedia Appendix 3 Calculations including forest and funnel plots.

10.2196/64856 Checklist 1 PRISMA (Preferred Reporting Items for Systematic Reviews and Meta-Analyses) for Abstracts checklist.

10.2196/64856 Checklist 3 PRISMA (Preferred Reporting Items for Systematic Reviews and Meta-Analyses) checklist.

## Abbreviations

DUT: Durban University of Technology
ES: effect size
FFE: face-to-face exercise
GRADE: Grading of Recommendations, Assessment, Development, and Evaluations
IREC: institutional research ethics committee
NEX: no exercise
OE: online exercise
PA: physical activity
PRISMA: Preferred Reporting Items for Systematic Reviews and Meta-Analyses
PROSPERO: International Prospective Register of Systematic Reviews
SMD: standard mean difference
T2D: type 2 diabetes
