# Supplementary material for: The Effectiveness of Online Exercise on Physical Activity, Motor Function, and Mental Health: Systematic Review and Meta-Analysis
Source: J Med Internet Res. 2025 Aug 15;27:e64856. doi: 10.2196/64856 (PMC12369988; doi:10.2196/64856)
Supplement: Multimedia Appendix 1 [file jmir-v27-e64856-s001.docx]

Search Strategy

A common search term was used across all 3 search engines

Search Term used WITH quotation marks:

“(exercise OR "PA") AND (internet* OR online* OR web* OR e-health OR digital OR tele* OR virtual) AND home*”

Search One

Filters and Limits:

The following filters were applied:

- Published journal articles
- Articles published from January 2020 to August 2022 (Search One), Articles published from August 2022 to February 2024 (Search Two)
- Articles published in English

| **Database** | **Search** | **Search Term** | **Results** |
| --- | --- | --- | --- |
| PubMed | 1 | (exercise OR "PA") AND (internet* OR online* OR web* OR e-health OR digital OR tele* OR virtual) AND home* | 3104 |
|  | 2 | (exercise OR "PA") AND (internet* OR online* OR web* OR e-health OR digital OR tele* OR virtual) AND home* | 808 |
| Cochrane | 1 | (exercise OR "PA") AND (internet* OR online* OR web* OR e-health OR digital OR tele* OR virtual) AND home* | 2512 |
|  | 2 | (exercise OR "PA") AND (internet* OR online* OR web* OR e-health OR digital OR tele* OR virtual) AND home* | 411 |
| Google Scholar | 1 | (exercise OR "PA") AND (internet* OR online* OR web* OR e-health OR digital OR tele* OR virtual) AND home* | 100 |
|  | 2 | (exercise OR "PA") AND (internet* OR online* OR web* OR e-health OR digital OR tele* OR virtual) AND home* | 100 |
