# Supplementary material for: The Effectiveness of Online Exercise on Physical Activity, Motor Function, and Mental Health: Systematic Review and Meta-Analysis
Source: J Med Internet Res. 2025 Aug 15;27:e64856. doi: 10.2196/64856 (PMC12369988; doi:10.2196/64856)

**Mental inactive control**

Depression

RVE: Hierarchical Effects Model

Model: Effect ~ 1

Number of clusters = 4

Number of outcomes = 5 (min = 1 , mean = 1.25 , median = 1 , max = 2 )

Omega.sq = 0.4223399

Tau.sq = 0.06983034

Estimate StdErr t-value dfs P(|t|>) 95% CI.L 95% CI.U

1 X.Intercept. 1.08 0.341 3.15 3 0.0512 -0.0102 2.16

Sig

1 *

---

Signif. codes: < .01 *** < .05 ** < .10 *

Anxiety

RVE: Hierarchical Effects Model

Model: Effect ~ 1

Number of clusters = 2

Number of outcomes = 2 (min = 1 , mean = 1 , median = 1 , max = 1 )

Omega.sq = 0

Tau.sq = 0.1026297

Estimate StdErr t-value dfs P(|t|>) 95% CI.L 95% CI.U

1 X.Intercept. 0.202 0.267 0.757 1 0.588 -3.19 3.59

Sig

1

---

Signif. codes: < .01 *** < .05 ** < .10 *

---

Mood/Emotion

RVE: Hierarchical Effects Model

Model: Effect ~ 1

Number of clusters = 5

Number of outcomes = 10 (min = 1 , mean = 2 , median = 2 , max = 3 )

Omega.sq = 0

Tau.sq = 0.08078837

Estimate StdErr t-value dfs P(|t|>) 95% CI.L 95% CI.U

1 X.Intercept. 0.473 0.153 3.09 4 0.0364 0.0486 0.897

Sig

1 **

---

Signif. codes: < .01 *** < .05 ** < .10 *

---

Mental well-being

RVE: Hierarchical Effects Model

Model: Effect ~ 1

Number of clusters = 4

Number of outcomes = 5 (min = 1 , mean = 1.25 , median = 1 , max = 2 )

Omega.sq = 1.439849

Tau.sq = 0

Estimate StdErr t-value dfs P(|t|>) 95% CI.L 95% CI.U

1 X.Intercept. 0.788 0.23 3.43 3 0.0417 0.0561 1.52

Sig

1 **

---

Signif. codes: < .01 *** < .05 ** < .10 *

---

Sleep

RVE: Hierarchical Effects Model

Model: Effect ~ 1

Number of clusters = 2

Number of outcomes = 3 (min = 1 , mean = 1.5 , median = 1.5 , max = 2 )

Omega.sq = 0.0003996312

Tau.sq = 0.04757327

Estimate StdErr t-value dfs P(|t|>) 95% CI.L 95% CI.U

1 X.Intercept. -0.255 0.182 -1.4 1 0.395 -2.57 2.06

Sig

1

---

Signif. codes: < .01 *** < .05 ** < .10 *

---

Self-Efficacy


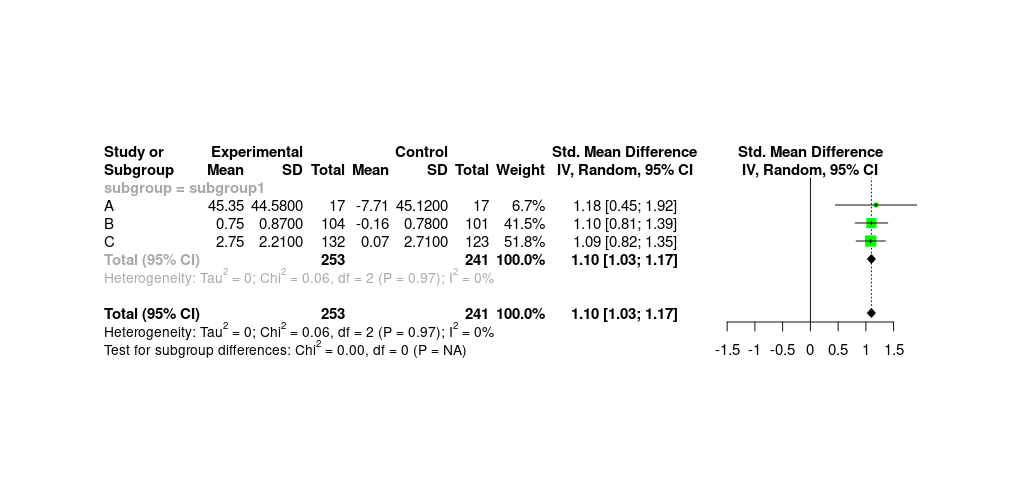


**Physical – inactive control**

Strength

RVE: Hierarchical Effects Model

Model: Effect ~ 1

Number of clusters = 5

Number of outcomes = 12 (min = 1 , mean = 2.4 , median = 2 , max = 5 )

Omega.sq = 0.05343153

Tau.sq = 0.1358272

Estimate StdErr t-value dfs P(|t|>) 95% CI.L 95% CI.U

1 X.Intercept. 0.605 0.198 3.06 4 0.0376 0.0562 1.15

Sig

1 **

---

Signif. codes: < .01 *** < .05 ** < .10 *

---

Endurance

RVE: Hierarchical Effects Model

Model: Effect ~ 1

Number of clusters = 5

Number of outcomes = 6 (min = 1 , mean = 1.2 , median = 1 , max = 2 )

Omega.sq = 0

Tau.sq = 0.3176633

Estimate StdErr t-value dfs P(|t|>) 95% CI.L 95% CI.U

1 X.Intercept. 0.848 0.307 2.76 4 0.0509 -0.00522 1.7

Sig

1 *

---

Signif. codes: < .01 *** < .05 ** < .10 *

---

Balance

RVE: Hierarchical Effects Model

Model: Effect ~ 1

Number of clusters = 4

Number of outcomes = 9 (min = 1 , mean = 2.25 , median = 1 , max = 6 )

Omega.sq = 0

Tau.sq = 0.1277684

Estimate StdErr t-value dfs P(|t|>) 95% CI.L 95% CI.U

1 X.Intercept. 0.523 0.146 3.57 3 0.0374 0.0573 0.988

Sig

1 **

---

Signif. codes: < .01 *** < .05 ** < .10 *

---

Gait

RVE: Hierarchical Effects Model with Small-Sample Corrections

Model: Effect ~ 1

Number of clusters = 2

Number of outcomes = 3 (min = 1 , mean = 1.5 , median = 1.5 , max = 2 )

Omega.sq = 0

Tau.sq = 0.03158021

Estimate StdErr t-value dfs P(|t|>) 95% CI.L 95% CI.U

1 X.Intercept. 0.261 0.196 1.33 1 0.41 -2.23 2.75

Sig

1

---

Signif. codes: < .01 *** < .05 ** < .10 *

---

Note: If df < 4, do not trust the results>

Flexibility

| RVE: Hierarchical Effects Model with Small-Sample Corrections  Model: Effect ~ 1  Number of clusters = 2  Number of outcomes = 4 (min = 2 , mean = 2 , median = 2 , max = 2 )  Omega.sq = 0  Tau.sq = 0.5252885  Estimate StdErr t-value dfs P(\|t\|>) 95% CI.L 95% CI.U  1 X.Intercept. 0.736 0.556 1.32 1 0.412 -6.32 7.8  Sig  1  ---  Signif. codes: < .01 *** < .05 ** < .10 *  ---  Note: If df < 4, do not trust the results> |
| --- |
|  |
| \| > \| \| --- \| |

Physical Activity

RVE: Hierarchical Effects Model

Model: Effect ~ 1

Number of clusters = 5

Number of outcomes = 7 (min = 1 , mean = 1.4 , median = 1 , max = 2 )

Omega.sq = 0

Tau.sq = 0.07154046

Estimate StdErr t-value dfs P(|t|>) 95% CI.L 95% CI.U

1 X.Intercept. 0.461 0.149 3.1 4 0.0364 0.0476 0.873

Sig

1 **

---

Signif. codes: < .01 *** < .05 ** < .10 *

Physical Function: Removed because of too much heterogeneity

**Physical – active control**

Strength

RVE: Hierarchical Effects Model

Model: Effect ~ 1

Number of clusters = 4

Number of outcomes = 14 (min = 1 , mean = 3.5 , median = 3.5 , max = 6 )

Omega.sq = 0.02213271

Tau.sq = 0.1255575

Estimate StdErr t-value dfs P(|t|>) 95% CI.L 95% CI.U

1 X.Intercept. -0.196 0.203 -0.965 3 0.406 -0.842 0.45

Sig

1

---

Signif. codes: < .01 *** < .05 ** < .10 *

---

Endurance

RVE: Hierarchical Effects Model

Model: Effect ~ 1

Number of clusters = 3

Number of outcomes = 4 (min = 1 , mean = 1.33 , median = 1 , max = 2 )

Omega.sq = 0

Tau.sq = 0

Estimate StdErr t-value dfs P(|t|>) 95% CI.L 95% CI.U

1 X.Intercept. -0.0363 0.0716 -0.507 2 0.663 -0.344 0.272

Sig

1

---

Signif. codes: < .01 *** < .05 ** < .10 *

---

Gait Speed

RVE: Hierarchical Effects Model with Small-Sample Corrections

Model: Effect ~ 1

Number of clusters = 2

Number of outcomes = 2 (min = 1 , mean = 1 , median = 1 , max = 1 )

Omega.sq = 0

Tau.sq = 0

Estimate StdErr t-value dfs P(|t|>) 95% CI.L 95% CI.U

1 X.Intercept. 0.252 0.000929 271 1 0.00235 0.24 0.264

Sig

1 ***

---

Signif. codes: < .01 *** < .05 ** < .10 *

---

Note: If df < 4, do not trust the results>

Blood Pressure

RVE: Hierarchical Effects Model with Small-Sample Corrections

Model: Effect ~ 1

Number of clusters = 5

Number of outcomes = 5 (min = 1 , mean = 1 , median = 1 , max = 1 )

Omega.sq = 0

Tau.sq = 0

Estimate StdErr t-value dfs P(|t|>) 95% CI.L 95% CI.U

1 X.Intercept. -0.0605 0.0587 -1.03 3.21 0.374 -0.241 0.12

Sig

1

---

Signif. codes: < .01 *** < .05 ** < .10 *

---

Note: If df < 4, do not trust the results>

Body Fat

RVE: Hierarchical Effects Model with Small-Sample Corrections

Model: Effect ~ 1

Number of clusters = 2

Number of outcomes = 2 (min = 1 , mean = 1 , median = 1 , max = 1 )

Omega.sq = 0

Tau.sq = 0

Estimate StdErr t-value dfs P(|t|>) 95% CI.L

1 X.Intercept. -0.385 0.0000681 -5654 1 0.000113 -0.386

95% CI.U Sig

1 -0.384 ***

---

Signif. codes: < .01 *** < .05 ** < .10 *

---

Note: If df < 4, do not trust the results>

Funnel plots

Strength vs. inactive


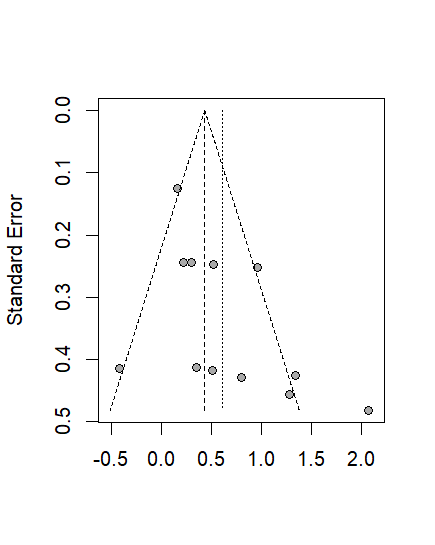


Mood/emotion vs. inactive


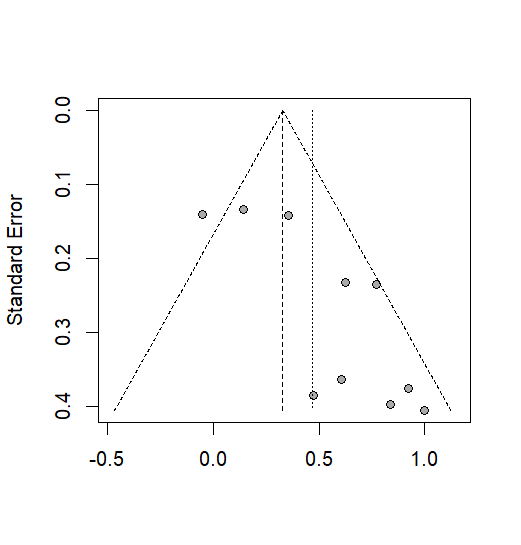


Strength vs active


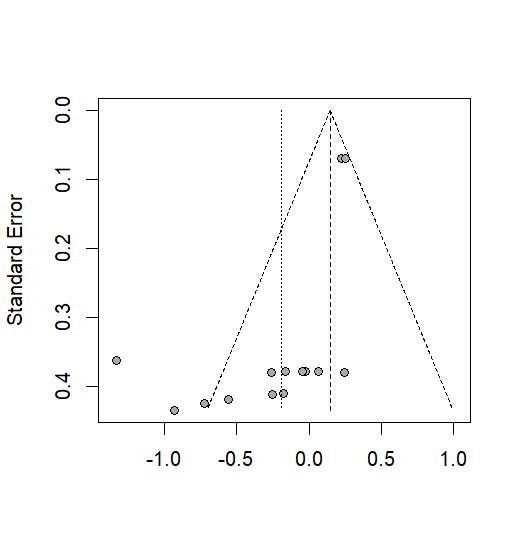

Supplement: Multimedia Appendix 3 [file jmir-v27-e64856-s003.docx]
